# Incarcération du périoste dans le cartilage de croissance dans une fracture-décollement épiphysaire: à propos d’un cas

**DOI:** 10.11604/pamj.2021.39.235.27764

**Published:** 2021-08-12

**Authors:** René Ayaovi Gayito Adagaba, Albéric Lionel Kolontchang Gatchou, Naima Loucheur, Jordan Boboe, Maturin Ganglo, Loriano Guedehounsou, Mahuklo Ulrich Brice Megninou, Pierre-Louis Docquier

**Affiliations:** 1 Service de Chirurgie Générale, Hôpital Saint Jean de Dieu de Tanguiéta, Tanguiéta, Bénin

**Keywords:** Décollement épiphysaire, périoste, enfant, rapport de cas, Epiphyseal separation, soft-tissue, child, case report

## Abstract

L´incarcération de corps étranger dans le cartilage de croissance est une pathologie rare. Elle survient souvent lors d´un décollement épiphysaire. Son diagnostic est radiologique, reposant sur l'imagerie par résonnance magnétique (IRM) cérébrale. Nous rapportons ici, le cas d´une fille de 13 ans qui s´est présenté pour un genou douloureux post traumatique gauche. L´examen clinique et les radiographies standards réalisées était en faveur d´un décollement épiphysaire type 1 de Salter-Harris. Le traitement de première intention qui avait consisté en une immobilisation plâtrée pendant trois semaines était non satisfaisante. Devant cet échec thérapeutique, une IRM a été réalisée et mise en évidence une incarcération de corps étranger dans le cartilage de conjugaison. La prise en charge secondaire a été chirurgicale avec des suites opératoires simples.

## Abstract (en)

Foreign body entrapment in growth plate cartilage is a rare disease. It often occurs in patients with epiphyseal separation. Its diagnosis is radiological, based on brain magnetic resonance imaging (MRI). We here report a case of a 13-year-old girl who presented with a painful left post traumatic knee. The clinical examination and the standard radiographs performed were in favor of a Salter-Harris type 1 epiphyseal detachment. The first-line treatment, which consisted of immobilization in a cast for three weeks, was unsatisfactory. Faced with this therapeutic failure, an MRI was performed and demonstrated an incarceration of a foreign body in the conjugation cartilage. Secondary management was based on surgery, without sequelae.

## Introduction

La complexité de la chondro-épiphyse qui est composée d'épiphyse, de cartilage de croissance et de gaine périchondrale n´est plus à démontrer [1, 2]. Cette zone charnière avec sa fragilité relative [3] est souvent source de lésion rare comme les incarcérations de corps étranger à la faveur d´un décollement épiphysaire [4]. La mise en évidence de ces incarcérations peut constituer un grand challenge car non vu sur des radiographies standards. L´imagerie par résonnance magnétique est l´examen de choix pour le diagnostic de telle lésion [5]. Le traitement reste chirurgical [6] avec comme principe: la désincarcération du corps étranger. La méconnaissance d´une telle lésion peut conduire à des séquelles irréversibles comme une épiphysiodèse [7]. Nous présentons ici le cas d'une jeune fille de 13 ans qui a présenté une fracture du décollement épiphysaire de fémur distal type 1 de Salter et Harris associée à une incarcération du périoste dans le cartilage de croissance, une association très rarement décrite dans la littérature.

## Patient et observation

**Information de la patiente:** il s´agit d´une fille de 13 ans, sans antécédents médicaux, qui s'est présentée pour une douleur et une impotence fonctionnelle du membre pelvien gauche à la suite d'un traumatisme fermé du genou gauche par un accident de la route.

**Résultats cliniques:** l´examen clinique était sans particularité et ne retrouvait qu´une tuméfaction douloureuse du genou gauche et un choc rotulien.

**Figure 1:**
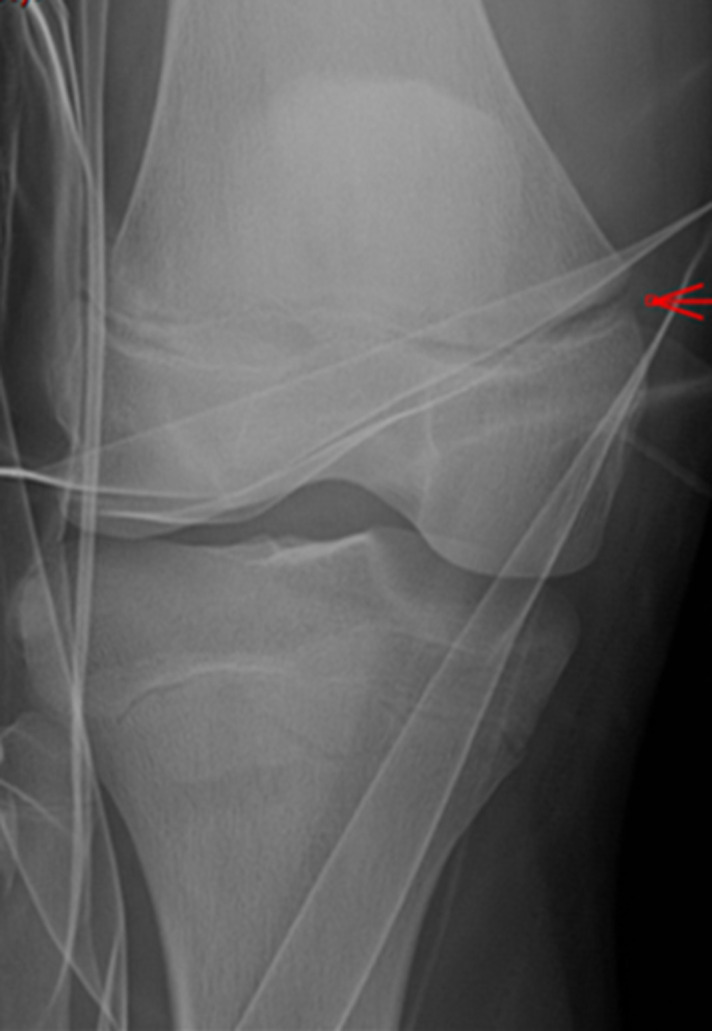
fracture-décollement épiphysaire type I Salter et Harris

**Démarche diagnostique:** les radiographies standards avaient mis en évidence une fracture décollement épiphysaire type 1 de Salter-Harris du fémur distal gauche (Figure 1).

**Figure 2:**
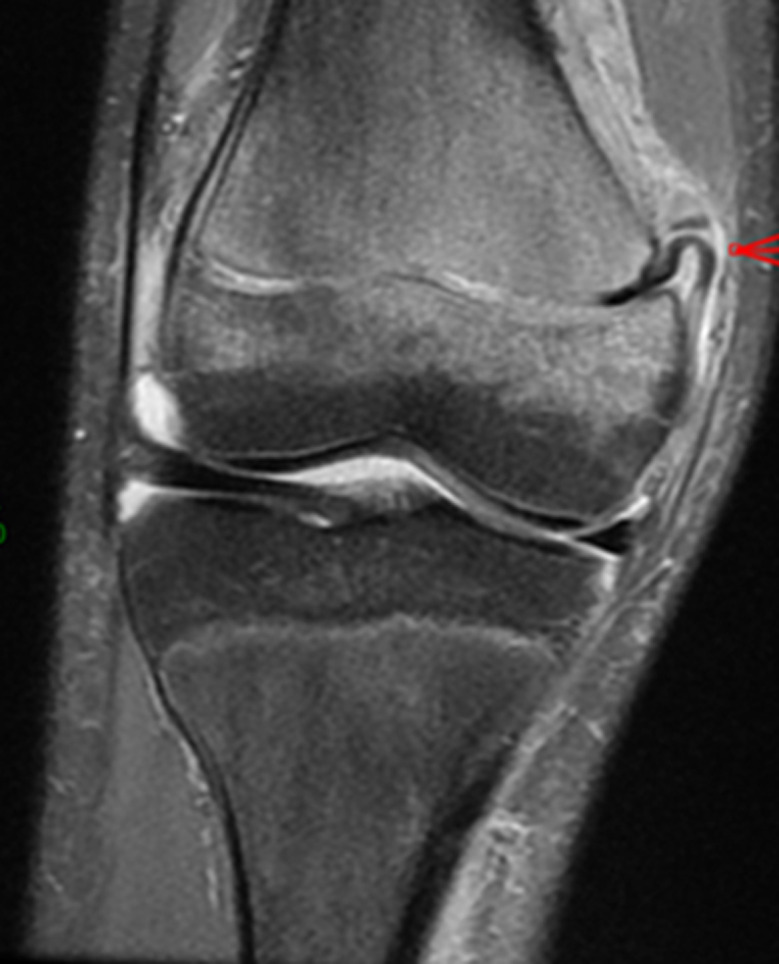
incarcération du périoste dans le cartilage de croissance vu à l´imagerie par résonance magnétique (IRM)

**Figure 3:**
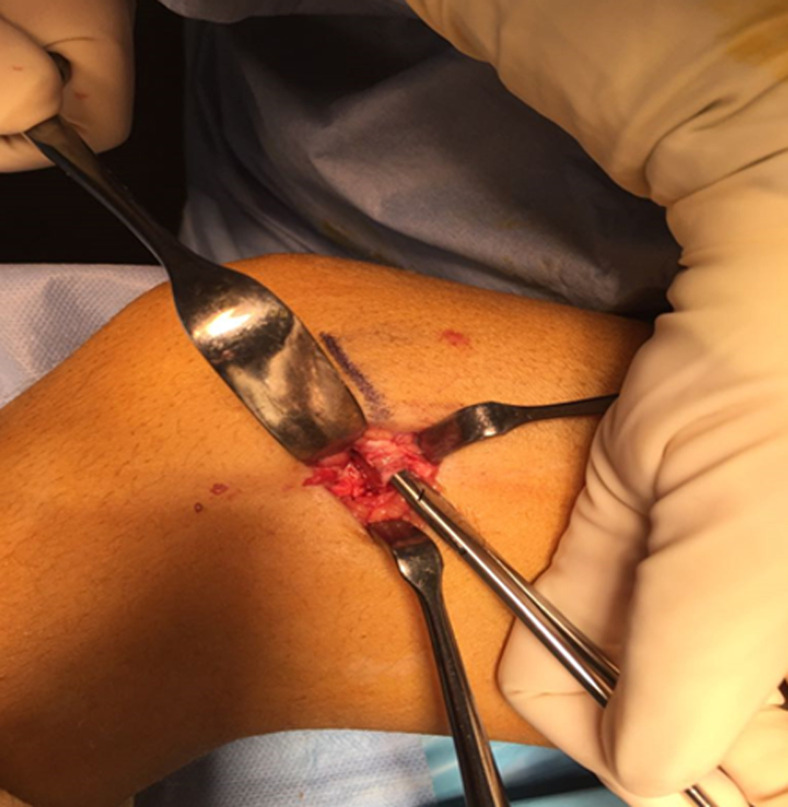
incarcération du périoste dans le cartilage de croissance vu en per-opératoire

**Intervention thérapeutique et suivi:** le traitement avait consisté en une immobilisation dans une attelle de Zimmer pendant 3 semaines. La patiente a été revue 3 semaines plus tard avec la persistance d´une boiterie à la marche. La clinique retrouvait une mobilisation passive et active du genou gauche douloureuse. Devant l´échec thérapeutique, il a été réalisé une IRM qui met en évidence une présence de corps étranger dans le cartilage de croissance du fémur distal gauche (Figure 2). L´indication chirurgicale fut posée en vue de l´extraction du corps étranger avec comme but: d´obtenir une réduction anatomique et d'éviter une épiphysiodèse. En per-opératoire, par un abord médial, on met en évidence une incarcération du périoste dans le cartilage de croissance. On réalise une désincarcération du périoste du cartilage de croissance (Figure 3) suivie d´une immobilisation du genou par une attelle en extension pendant 3 semaines. Les suites opératoires ont été simples. La patiente est sortie au 3^e^ jour post-opératoire. Elle a été revue 6 semaines après l'intervention sans aucune plainte et avec une reprise complète des activités sportives.

## Discussion

Le cartilage de croissance est une zone charnière située entre l'épiphyse et la métaphyse, avec une fragilité relative [3]. Par conséquent, une fracture décollement épiphysaire [4] peut être préjudiciable au pronostic fonctionnel du membre pelvien chez l´enfant. Les données épidémiologiques montrent que 15 à 30% de toutes les lésions ostéoarticulaires chez l'enfant intéressent la région épiphysaire. Environ 80% de ces lésions épiphysaires surviennent entre 10 et 16 ans, avec un pic d'âge pour les décollements épiphysaires entre la 12^e^ et la 13^e^ année de vie [8]. Un pourcent (1%) des fractures-décollements épiphysaires se produisent dans le fémur distal [9]. Ceci est important dans la mesure où le taux de complications pour les fractures distales du fémur serait de 50% dans certaines séries avec comme complication à haut risque, une épiphysiodèse.

De nombreuses raisons expliquant le taux élevé d´épiphysiodèse dans les fractures du fémur-décollement épiphysaires de Salter et Harris [10], telles que: la grande énergie absorbée par l'os lors des traumatismes (pouvant nuire à la circulation sanguine épiphysaire et zone germinale), la configuration ondulante de la plaque de croissance fémorale distale, entraînant davantage de blessures par cisaillement des cellules de réserve, le fait que beaucoup de ces traumatismes surviennent chez des adolescents en fin de croissance, le degré de déplacement de la fracture et le préjudice supplémentaire causé par le geste chirurgical. Récemment, l´incarcération du périoste dans le cartilage de croissance a été considéré par certains comme une cause prédisposante à l´épiphysiodèse [7].

Les radiographies du genou sont généralement l'examen paraclinique de première intention suite à un traumatisme du fémur distal. Dans notre cas clinique, la fracture-décollement épiphysaire du fémur Salter-Harris type 1 était évidente sur le plan radiographique (Figure 1). Mais ni la clinique ni la radiographie ne pouvait faire suspecter une lésion associée. L'IRM a été extrêmement utile dans la mise en évidence de cette association lésionnelle qu´est l´incarcération du périoste dans le cartilage de croissance. En effet, l'imagerie par résonance magnétique offre une excellente visualisation du cartilage de croissance et joue un rôle important dans l'évaluation des lésions associées en cas de décollements épiphysaires [5].

Dans une grande série de 315 enfants ayant présentés un traumatisme du genou, 9 patients (2,9%) avaient une fracture décollement épiphysaire, la lésion de Salter Harris de type 2 étaient la plus courante [5]. Parmi ces 9 enfants, 6 avaient une lésion associée. Dans les fractures décollement épiphysaire Salter-Harris type 1, il existe une séparation épiphysaire complète sans fracture osseuse adjacente [11]. À notre connaissance, le premier cas d´incarcération du périosté dans le cartilage de croissance intéressant le fémur distal a été rapporté en 2011 [7]. Dans cette publication, les auteurs ont signalé deux cas. En 2015, un autre cas avait été publié [6]. Il s´agissait pour ces 3 premiers cas publiés dans la littérature d´une association avec une fracture décollement épiphysaire type 2 de Salter-Harris.

L´originalité de notre cas est qu´il diffère des autres par l´association encore plus rare de l´incarcération du périoste dans le cartilage de croissance avec une fracture décollement épiphysaire type 1 de Salter et Harris. L´incarcération du périoste dans le cartilage de croissance nécessite dans la plupart des cas une intervention chirurgicale, car il peut être une cause d´épiphysiodèse. Dans le cas présenté, la chirurgie pouvait être discutée car la fillette avait 13 ans, donc en fin de croissance presque. Le risque d´épiphysiodèse était donc faible.

## Conclusion

L´incarcération du périoste dans le cartilage de croissance chez l´enfant reste une entité rare. Elle survient à la faveur d´un décollement épiphysaire et plus fréquemment cité dans des grades sévères. La clinique est souvent trompeuse et l´IRM reste l´examen de choix pour le diagnostic. La prise en charge doit être chirurgicale afin de redonner à l´articulation concernée son autonomie. Notre cas plus rare, reste original de par le degré moindre du décollement épiphysaire (type 1 de Salter et Harris). Il exige de la part de tout praticien de la méfiance devant toute fracture décollement épiphysaire.

## References

[1] Salter RB, Harris WR (1963). Injuries involving the epiphyseal plate. J Bone Joint Surg.

[2] Arkader A, Warner WC, Horn BD, Shaw RN, Wells L (2007). Predicting the outcome of physeal fractures of the distal femur. J Pediatr Orthop.

[3] Smith DG, Geist RW, Cooperman DR (1985). Microscopic examination of a naturally occurring epiphyseal plate fracture. J Pediatr Orthop.

[4] Jaramillo D, Shapiro F, Hoffer FA (1990). Posttraumatic growth-plate abnormalities: MR imaging of bony-bridge formation in rabbits. Radiology.

[5] Close BJ, Strouse PJ (2000). MR of physeal fractures of the adolescent knee. Pediatr Radiol.

[6] Chen J, Abel MF, Abel Fox MG (2015). Imaging appearance of entrapped periosteum within a distal femoral Salter-Harris II fracture. Skeletal Radiol.

[7] Segal LS, Shrader MW (2011). Periosteal entrapment in distal femoral physeal fractures: harbinger for premature physeal arrest?. Acta Orthop Belg.

[8] Celareka A, Fischerauerb SF, Weinbergb AM, Tschegga EK (2014). Fracture patterns of the growth plate and surrounding bone in the ovine knee joint at different ages. J Mech Behav Biomed Mater.

[9] Dahl WJ, Silva S, Vanderhave KL (2014). Distal femoral physeal fixation: are smooth pins really safe?. J Pediatr Orthop.

[10] Basener CJ, Mehlman CT, DiPasquale TG (2009). Growth disturbance after distal femoral growth plate fractures in children: a meta-analysis. J Orthop Trauma.

[11] Nancy Chauvin MD, Diego Jaramillo (2012). Occult distal femoral physeal injury with disruptionof the perichondrium. J Comput Assist Tomogr.

